# Molecular Recognition by Pillar[5]arenes: Evidence for Simultaneous Electrostatic and Hydrophobic Interactions

**DOI:** 10.3390/pharmaceutics14010060

**Published:** 2021-12-28

**Authors:** Borja Gómez-González, Luis García-Río, Nuno Basílio, Juan C. Mejuto, Jesus Simal-Gandara

**Affiliations:** 1 Departamento de Química Física, Facultade de Química, Universidade de Santiago de Compostela, 15782 Santiago, Spain; gomezgonzalezborja@gmail.com; 2 Laboratório Associado Para a Química Verde (LAQV), Rede de Química e Tecnologia (REQUIMTE), Departamento de Química, Faculdade de Ciências e Tecnologia, Universidade NOVA de Lisboa, 2829-516 Caparica, Portugal; nuno.basilio@fct.unl.pt; 3 Department of Physical Chemistry, Faculty of Science, University of Vigo, 32004 Ourense, Spain; 4 Nutrition and Bromatology Group, Analytical and Food Chemistry Department, Faculty of Food Science and Technology, University of Vigo, 32004 Ourense, Spain; jsimal@uvigo.es

**Keywords:** pillararene, host:guest, supramolecular, hydrophobic, ITC, NMR

## Abstract

The formation of inclusion complexes between alkylsulfonate guests and a cationic pillar[5]arene receptor in water was investigated by NMR and ITC techniques. The results show the formation of host-guest complexes stabilized by electrostatic interactions and hydrophobic effects with binding constants of up to 10^7^ M^−1^ for the guest with higher hydrophobic character. Structurally, the alkyl chain of the guest is included in the hydrophobic aromatic cavity of the macrocycle while the sulfonate groups are held in the multicationic portal by ionic interactions.

## 1. Introduction

Supramolecular chemistry is a topic of great interest to the scientific community that wants to take advantage of non-covalent interactions, such as van der Waals forces, hydrogen bonds, π-π stacking interaction, electrostatic interactions, or hydrophobic/hydrophilic interactions, with the aim of implementing and explaining increasing complexity systems (bottom-up approach) [1,2,3]. During the last decades, numerous supramolecular systems have been successfully developed and in the literature, there are numerous investigations regarding their applications as functional materials, in catalytic processes, electronic devices, sensors, or drug carriers, etc., [4,5,6]. Among these applications, nanomedicine presents a promising potential for modernizing traditional biomedical practices, and in this context, the design of new supramolecular systems in the nanometric range is one of the new frontiers that will offer new diagnostic and therapeutic applications in the field of nanomedicine (drug delivery, gene delivery, drug/gene co-delivery, bioimaging or photodynamic therapy) [7,8].

Noncovalent interactions present several advantages in comparison to covalent ones:

(i) An easy and facile approach for building supramolecular structures avoiding synthetic processes [9].
(ii) Supramolecular methods are cost-effective and environmentally friendly.
(iii) Supramolecular materials consist of components connected by non-covalent interactions and experiencing spontaneous assembly and disassembly processes [10,11,12].
(iv) The formation of supramolecular materials is reversible and capable of being recycled and self-repaired from external mechanical damage.
(v) Supramolecular materials have the ability to respond to external stimuli being able to rearrange their structures or morphologies toward more stable states. This adaptive capability can be utilized for the development of stimuli-responsive functional materials [13,14,15,16].
(vi) In addition, it allows the manipulation of supramolecular molecules or building blocks at the molecular level, modulating sizes and morphologies using the “bottom-up” method, providing a variety of novel diagnostic and therapeutic platforms toward applications in nanomedicine.

Within the different non-covalent interactions considered as supramolecular phenomena, the host:guest between different substrates and macrocycles have been studied extensively in recent decades. By including host:guest, two or more molecules can be integrated in a simple and reversible way. This offers us multiple possibilities for new supramolecular structure design. Molecular recognition that involves host:guest interactions play a vital role in life-sustaining biological processes [17,18]. Macrocyclic compounds have been extensively used and intensively investigated as prime host receptors with high affinity and selectivity for complementary small guest molecules or ions. Examples of macrocycles include cryptands [19], crown ethers [20,21,22], cyclophanes [23], cyclopeptides [24,25,26], cyclodextrins [27,28,29], resorcinarenes [30], cucurbit[n]urils [31,32,33,34], calix[n]arenes [35,36,37,38], and pillar[n]arenes [39]. These macrocycles (hosts) have cavities that allow the encapsulation of substrates of interest (guest). The external properties exhibited by the host molecules favor interaction with the surrounding solvent. On the other hand, the characteristics of the cavities allow the inclusion of the guest. This inclusion occurs through different causes (hydrophobic and/or electrostatic interactions, formation of hydrogen bonds, suitable molecular shape and/or size, etc. In fact, encapsulation in an aqueous solution of hydrophobic guest molecules in macrocyclic hydrophobic cavities is one of the most common cases. The host:guest complex will exhibit high stabilities, providing robust and reliable structures for obtaining supramolecular systems in aqueous media.

Pillar[n]arenes are one of the most recent families of macrocyclic hosts used in supramolecular chemistry [40]. Pillararenes bring together some interesting characteristics of other host systems in a single molecular structure, such as a highly symmetrical pillar-shaped structure which is similar in many respects to that of highly symmetrical cucurbiturils, a π-rich aromatic cavity, also found in calixarenes, and several hydroxyl moieties on both rims, a feature shared with the highly functionalized cyclodextrins. Substituents on both rims of pillararenes affect their physical properties, such as solubility, conformational and host:guest properties. Pillar[n]arenes are very useful structures useful for the design of different supramolecular systems [41,42,43,44,45,46,47,48,49,50,51]. In particular, these systems have interrelated applications in the biomedical and pharmacological fields as drug carriers [46], transmembrane channels [47], or cellular glue [48].

**Scheme 1:**
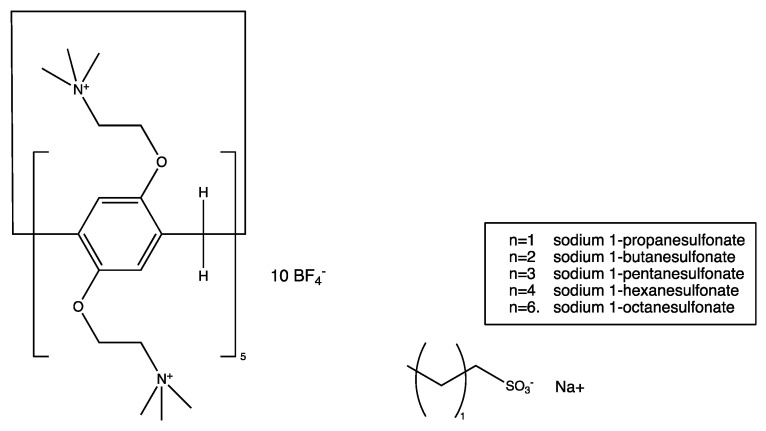
Cationic pillar[5]arene and alkylsulfonates.

Another aspect to consider is that the presence of charged groups on the pillars[n]arenes convert them into water-soluble substrates (see Scheme 1). Furthermore, its ability to incorporate a wide variety of hosts into its cavity [52,53], its applications as catalysts [54,55,56], detection [57,58] and gene nanocarriers [59] in aqueous media, has caused that numerous investigations focus on them. When oppositely charged molecules were evaluated as hosts, electrostatic interactions contribute significantly to stabilizing the resulting supramolecular system.

Pillararenes-based host-guest systems comprising amphiphilic guests offer interesting strategies for the development of novel stimuli-responsive drug-delivery systems which improve precision and efficiency in drug delivery [60,61,62,63,64,65,66].

In this sense, knowledge of the driving forces behind the host:guest complex formation process is important. We know about the electrostatic interactions between the charged groups of the pillar[5]arene and the ionic substrates, however, the role played by hydrophobic/hydrophilic interactions must be explored in detail. In this context, fundamental studies on the interaction between charged pillararene receptors and model amphiphilic compounds are of utmost importance for the intended pharmaceutical applications in relation to these macrocycles.

In this article, we present a structural and thermodynamic study on the host-host complexes between a cationic pillar[5]arene and charged amphiphilic compounds, which by keeping the head group constant, its hydrophobicity can be modulated by modifying the length of the chain hydrocarbon. The family of amphiphiles chosen was the alkylsufonates (see Scheme 1).

## 2. Materials and Methods

### 2.1. Materials

The highest purity commercially available reagents were supplied by Sigma-Aldrich (Madrid, Spain) and were used without further purification. The water-soluble cationic pillar[5]arene was obtained by a synthetic procedure described elsewhere [67]. Br- exchange by BF4- was carried out as follows: To a solution with Br- (1.17 g, 0.514 mmol) in Milli-Q water at room temperature and with stirring, AgBF4 was added slowly in little portions. A grayish precipitate was obtained. The suspension was centrifuged, and supernatant was collected and filtered (0.45 µm). A yellowish solid was obtained after removing the solvent (1.15 g, 96%). ^1^H NMR (D_2_O, 300 MHz): δ = 6.89 (s, 10H); δ = 4.36 (s, 20H); δ = 3.91 (s, 10H); δ = 3.72 (s, 20H); δ = 3.19 (s, 90H); ^13^C NMR (D_2_O, 75 MHz): δ = 149.2 (C, 10C); δ = 129.8 (C, 10C); δ = 115.9 (CH, 10C); δ = 64.8 (CH_2_, 10C); δ = 62.3 (CH_2_, 10C); δ = 53.7 (CH_3_, 30C); δ = 29.3 (CH_2_, 5C); MS (ESI): m/z calcd for [TMAP5^10+^.9BF_4_^−^]^2+^ 2253.4; found 2253.2; calcd for [TMAP5^10+^.8BF_4_^−^]^2+^ 1083.3; found 1083.1. The final product was analyzed by thermal gravimetric analysis to assess volatile content.

### 2.2. Microcalorimetry

An isothermal titration microcalorimeter (VP-ITC) supplied by Microcal Co. (Northamptoh, MA, USA) at 1 atm and 25 °C to carry out the microcalorimetric titrations. The procedure used for each titration consisted in sequentially injecting a guest solution in a syringe (0.270 mL) with shaking (459 rpm) into a host solution in the sample cell (1.459 mL). Before each titration, the samples were degassed and thermostatted using an accessory supplied by ThermoVac (Leybold Hispánica, Barcelona, SPAIN). For the reference cell, the same sample was used as in the sample cell. The first injection was discarded in all the experiments carried out in order to suppress the diffusion effects in the calorimetric cell of the syringe material. The number of injections, their volume and the spacing time between each one were varied according to the experiment. Binding constants were calculated from the titration curve by using the AFFINImeter software (S4SD, Santiago de Comostela, SPAIN).

### 2.3. NMR Spectrometry

NMR experiments were conducted at 25 °C on a spectrometer supplied by Bruker (Bruker NEO 17.6 T) (Billerica, MA, USA) with 750 MHz proton resonance, equipped with a ^1^H/^13^C/^15^N triple resonance PA-TXI probe with deuterium lock channel and shielded PFG z-gradient. The control software was TopSpin 4.0. Chemical shifts were referenced to the lock deuterium solvent. Spectra have been processed and analyzed using Mestrenova software v14.0 supplied by Mestrelab Inc (Santiago de Compostela, SPAIN). The 1D ^1^H spectrum has been measured with 128 scans, d1 2s relaxation delay and 2.75 s FID acquisition time (aq). The FID has been acquired with 64k complex data points. It has been processed using Fourier Transformation (FT) and zero-filling. 131 k data points spectra have been obtained. The total measurement time was ~10 min.

A two-dimensional 2D COSY spectrum magnitude mode was measured (pulse sequence “cosygpppqf” of Bruker library). The relaxation delay (d_1_) and the FID acquisition time (at) were 2 and 0.172 s, respectively. The spectrum was measured with eight scans. The number of points in the direct and indirect dimensions was 4 k and 160, respectively. The spectrum was processed with apodization with a sine-bell function in both dimensions and represented in the magnitude mode. The total measurement time was ~48 min.

A two-dimensional 2D HSQC multiplicity edited 1H-13C spectrum was measured (pulse sequence “hsqcedetgpsisp 2.4” of the Bruker library). The spectrum includes adiabatic inversion pulses in ^13^C and suppression of COSY type artifacts. The INEPTs transfers were optimized for a nominal value of ^1^J_CH_ of 145 Hz. The delay for multiplicity selection was set to 1/(2 · ^1^J_CH_) to detect with the same sign signals of CH_3_ and CH groups and with opposite phase CH_2_ groups. The relaxation delay (d_1_) and the FID acquisition time (at) were 1.6 and 0.112s, respectively; 2048 and 160 complex points in the t2 and t1 dimensions spectrum were acquired. Scans number per t1 increment was 8. The total measurement time was ~1 h 15 min.

## 3. Results

The hydrophobic cavity of the pillar[5]arene, together with the presence of five positive charges in each rim makes this macrocycle an excellent receptor for amphiphilic anionic guests. The complexation of the different alkylsulfonates, whose hydrophilic head exhibits a negative charge, (G) by the pillar[5]arene (H) was studied by different experimental techniques.

### 3.1. NMR Evidence of N-Octylsulfonate Complexation by Pillararene

**Figure 1:**
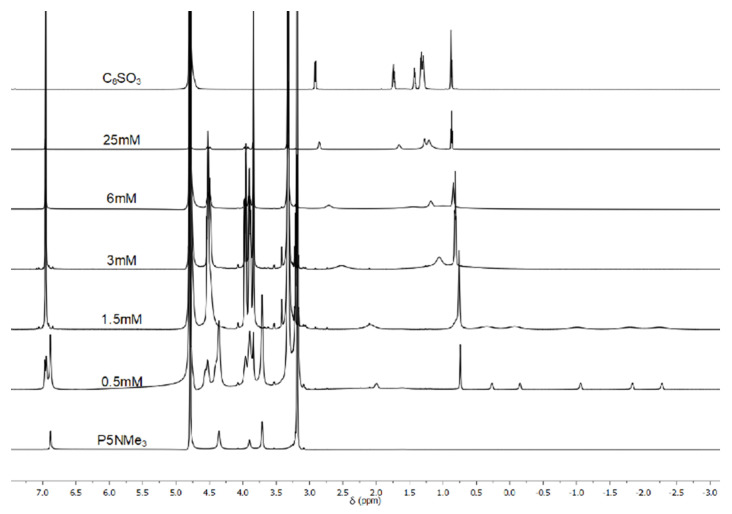
^1^H NMR spectra in D_2_O at 25 °C for pillararene (1.5 mM); octylsulfonate (1.5 mM) and mixtures of both with a constant concentration of pillararene (1.5 mM) and different concentrations of octylsulfonate.

NMR spectroscopy has been used to determine the structures of macrocycles complexes. The ^1^H NMR spectra of octylsulfonate upon mixing in different proportions with pillararene can be observed in Figure 1.

**Figure 2:**
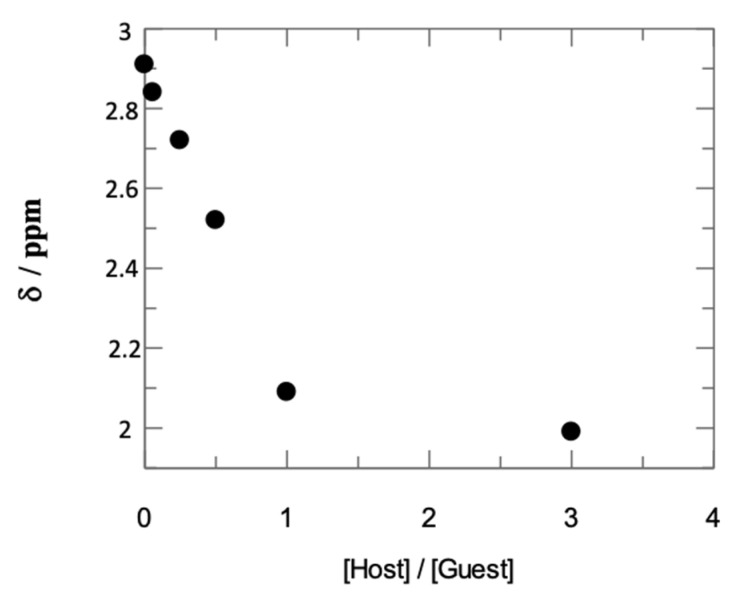
Chemical shifts for hydrogen atoms in alpha position to the sulfonate group in octylsulfonate in the presence of increasing concentrations of host.

All protons of octylsulfonate appear upfield-shifted with respect to the free guest upon addition of pillararene, indicating that an inclusion complex was formed. These results indicate that octylsulfonate is incorporated into the magnetic shielding region of the pillararene aromatic cavity with the sulfonate group pointing towards the trimethylammonium groups of the host. Moreover, the host proton signals are also affected by complexation due to the asymmetric structure of the guest and the manner in which it is inserted into the host cavity [52]. To determine the binding stoichiometry of the host:guest complex, considering that fast exchange on the NMR chemical shift timescale was observed for this complex, an NMR titration at constant host concentration, was carried out. Figure 2 shows that the magnitude of the upfield shift for guest hydrogen atoms increases upon a gradual increase of the [host]/[guest] ratio, reaching a plateau for values higher than 1, indicating a 1:1 stoichiometry for the inclusion complex.

**Figure 3:**
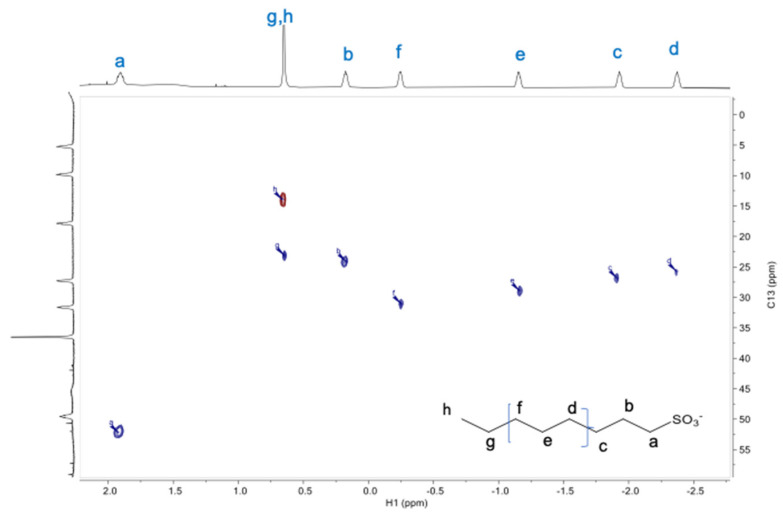
HSQC spectra for a mixture of 1.5 mM of Pillararene and 0.5 mM of octylsulfonate in D_2_O a 25 °C. Labels for octylsulfonate hydrogen atoms are according to the picture shown in the figure.

**Figure 4:**
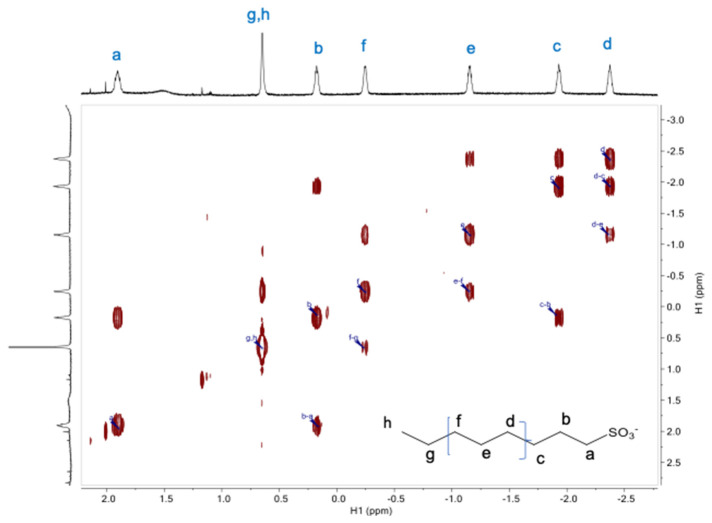
COSY spectra for a mixture of 1.5 mM of Pillararene and 0.5 mM of octylsulfonate in D_2_O a 25 °C. Labels for octylsulfonate hydrogen atoms are according to the picture shown in the figure.

Detailed analysis of spectrum for [Pillararene] = 1.5 mM and [octylsulfonate] = 0.50 mM (Figure 1) reveals that the signal corresponding to the methylene groups in positions C4–C7 of octylsulfonate splits into different signals, allowing a clear characterization of the inclusion complex. Figure 3 and Figure 4 show the HSQC and COSY spectra respectively allowing the assignment of all signals in the NMR spectrum.

**Figure 5:**
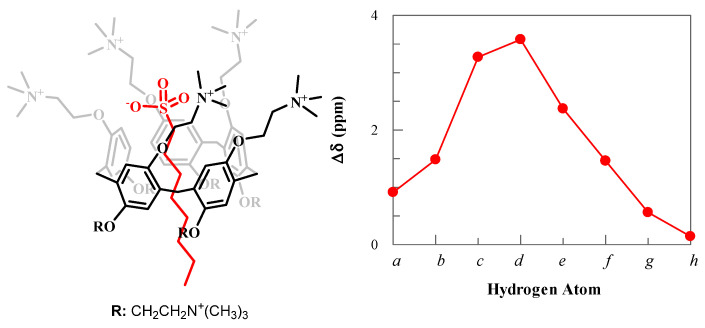
(**Left**) Plot of the magnitude of complexation induced chemical shift, Δδ, as a function of the hydrogen atom position (starting at the sulfonate group). (**Right**) Schematic picture of the host:guest complex showing hydrogens Hc and Hd fully incorporated into the aromatic region of the host. For simplicity only two trimethylammonium groups of pillararene are shown.

Assignment of NMR signals allows us to quantify the magnitude of the complexation-induced upfield effect for each hydrogen atom in octylsulfonate (results showed in Table 1). We refer to a complexation-induced chemical shift as the difference between the chemical shift observed for the guest free and complexed, Δδ = δ_free_ − δ_bound_. The magnitude of Δδ is dependent on the hydrogen atom position along the alkyl chain of octylsulfonate. It is remarkable the very large magnitude of the upfield effects with values larger than Δδ = 3 ppm for some central chain nuclei. Hydrogen atoms Hc and Hd show the large Δδ values allowing to propose a structure for the host:guest complex as shown in Figure 5. Hydrogen atoms in positions c and d are located inside the aromatic region of the pillararene allowing the large Δδ values, Δδ > 3 ppm. Hydrogens at position e should be just below this region but close to the aromatic groups (Δδ = 2.3 ppm). It is remarkable that hydrogen atoms at positions g and h (Δδ < 1 ppm), as well as in the alpha position to the sulfonate group, are clearly located outside the aromatic region.

**Figure 6:**
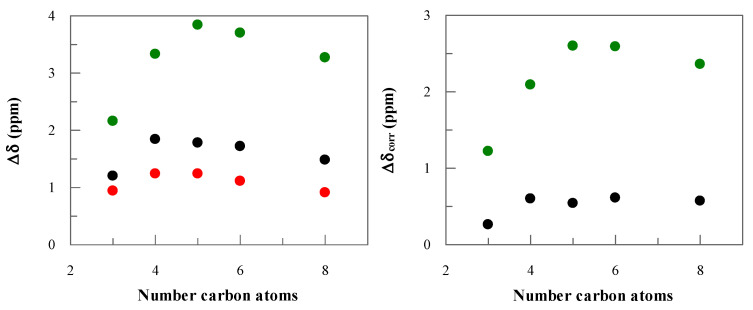
(**Left**) Influence of the number of carbon atoms in the alkyl chain of alkylsulfonate on the chemical induced upfield effect, Δδ: (●) Ha; (●) Hb and (●) Hc. (**Right**) Values of upfield effect for hydrogen atoms in positions (●) Hb and (●) Hc after correction by upfield effect of hydrogens Ha.

Similar experiments were conducted for shorter chain alkylsulfonates with three to six carbon atoms (see Table 1) revealing that hydrogen atoms in positions Hc and Hd show the large upfield effects confirming that these atoms are clearly included inside the pillararene cavity. The complexation picture shows the sulfonate aligned with the trimethylammonium head groups of the receptor in such a way that electrostatic interaction should be the major driving force for complexation. It is remarkable that Δδ values are also dependent on the nature of the alkylsulfonate (see Figure 6). In fact, Ha hydrogen atoms show the large upfield effect for alkylsulfonates with four and five carbon atoms, meanwhile, alkyl sulfonates with three and eight carbon atoms present smaller values. On the other hand, hydrogens Hc show the large upfield effect for C_5_SO_3_^−^ and C_6_SO_3_^−^, and hydrogens Hb show the large Δδ for C_4_SO_3_^−^ and C_5_SO_3_^−^. More clearly, Figure 6-left shows that the magnitude of Δδ is strongly dependent on the number of carbon atoms in the alkylsulfonate for hydrogens Hc > Hb > Ha, being indicative of a different degree of penetration into the pillararene cavity. Figure 6-right represents normalized Δδ_corr_ by subtracting the values corresponding to hydrogens Ha. The normalized values are directly comparable and indicate that Hc hydrogens are much closer to the cavity than Hb and that an optimal degree of penetration is reached for five atoms of carbon. Alkylsulfonates with three and four carbon atoms can form external complexes where the carbon atoms do not fit neatly together. This causes that the magnitude of Δδ_corr_ does not reach an optimal value. Likewise, it is observed that for octylsulfonate, the Hc hydrogens present a lower inclusion than for the 5 carbon atom homolog. This behavior may be due to a hydrophobic pushup effect that compels the sulfonate group towards a plane superior to the portal of the pillararene in order to accommodate more methylene groups inside the cavity. At the same time, the possibility that the hydrophobic effect induces a greater degree of folding of the alkyl chain in order to maximize the number of carbon atoms that can be included in the cavity should be considered.

These results indicate that the location of the sulfonate group should be dependent on the number of carbon atoms, being closer to the positive portal of the pillararene for C_5_SO_3_^−^ and C_4_SO_3_^−^. This behavior can be observed for hydrogen atoms in positions Hb and Hc, being clear evidence of a different degree of guest penetration into the host cavity and, consequently, ruling out the electrostatic attraction as the only interaction stabilizing the host:guest complex.

### 3.2. Calorimetric Titrations for Alikylsulfonate Recognition by Pillararene

**Figure 7:**
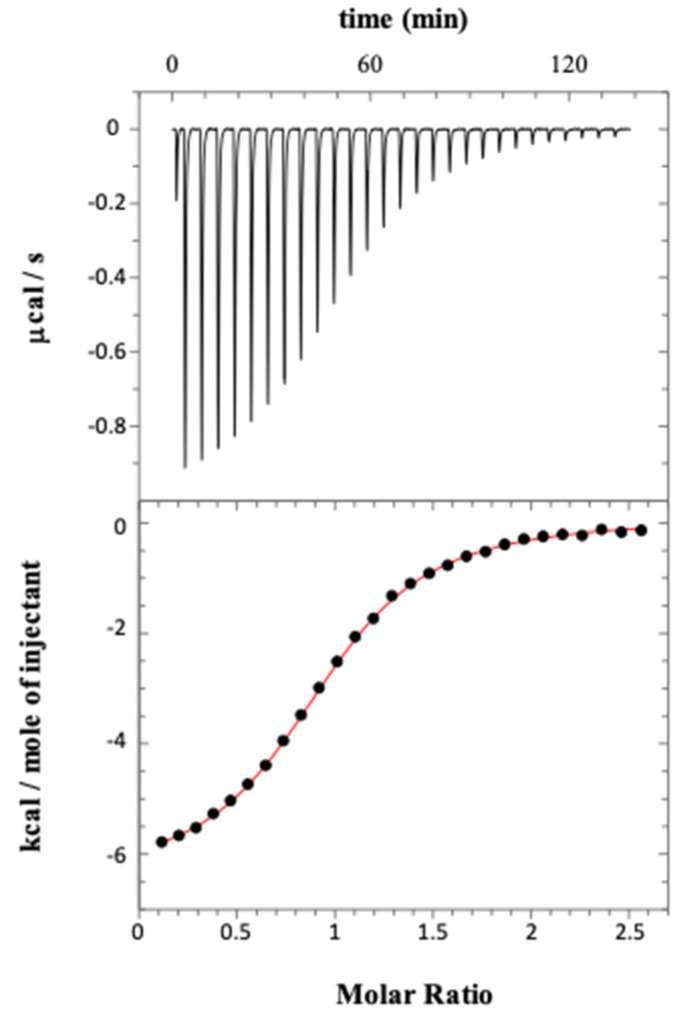
Microcalorimetric titration of butylsulfonate (G) with pillar[5]arene (H) in water at 25 °C. (**Top**): Raw data for the 28 sequential injections (10 μL per injection) of a solution of G (0.5 mM) into a solution of H (0.04 mM). (**Bottom**): “Net” heat effects fitted using the “one set of sites” binding model.

In order to quantitatively evaluate the complexation of pillar[5]arene with each guest and the stoichiometry of the complex formed, an isothermal calorimetry titration was carried out at 25 °C under neutral conditions. Each titration was done by consecutively adding the guest to the host in the sample cell. As an example, each butylsulfonate titration in the sample cell containing the pillar[5]arene is shown in Figure 7 (see Supplementary Materials for other alkylsulfonates). The experimental data were satisfactorily fitted to a model of “a set of binding sites”, obtaining the binding constant (K) and the thermodynamic parameters (Table 2).

The results indicate that complexation is mainly enthalpy-driven (ΔH^0^ = −(6.60 ± 0.01) kcal/mol) accompanied by favorable entropic changes (TΔS^0^ = 2.12 kcal/mol), this balance is more favorable to the enthalpic term with the other alkylsufonates.

From the results of the experiments obtained for guests and other macrocyclic compounds, it has been shown that non-covalent interactions contribute to enthalpic changes, while entropy changes can be attributed to conformational changes and/or effects associated with desolvation processes [68]. Thus, hydrophobic or electrostatic interactions, together with dehydration processes, have a positive contribution to entropy. The negative contribution would be produced by the loss of conformational freedom degrees (both on the guest and on the host). Thus, the values obtained for the thermodynamic parameters would indicate that the electrostatic interactions, π-π, and C-H⋯π interactions between the aromatic ring and the methyl group of the alkylsulfonate and the electron-rich pillararene cavity would give rise to a favorable contribution on enthalpy. At the same time, the solvent molecules (water) that surround both the host and the guest are released into the bulk water and would be the cause of the entropic increase. The binding constant obtained, K = (2.63 ± 0.01) × 10^6^ M^−1^, is comparable with those reported for negatively charged pillararenos [52,53,68,69] or calixarenes [70,71,72].

Experimental results reported in Table 2 show alkylsulfonate binding constants to be very sensitive to alkylsulfonate chain length with an increase of almost 10^4^ fold ongoing from propane to octanesulfonate. Quantitative analysis of these binding constants requires correction of binding constant for propanesulfonate. Because of its smaller value, experimental results were obtained in the presence of [Pillararene] = 0.25 mM instead of [Pillararene] = 0.04 mM used for other alkylsulfonates. Previous results from our group have shown that toluenesulfonate binding constant to pillararene decreases from 1.37 × 10^6^M^−1^ to 3.18 × 10^4^M^−1^ by increasing the host concentration from 0.01 to 0.1 mM [53]. This behavior is due to BF_4_^−^ complexation by the pillararene, which difficult the entrance of the guest. Extrapolation to alkylsulfonates implies that propanesulfonate binding constant of 1.86 × 10^5^ M^−1^ should be used for comparative proposes.

**Figure 8:**
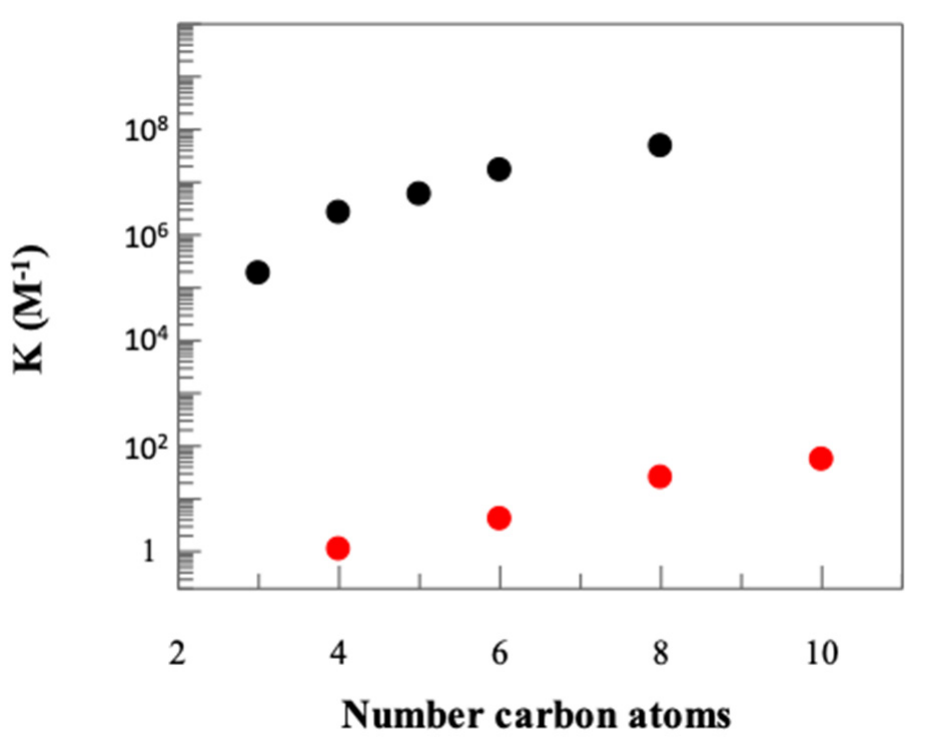
(●)Influence of alkyl chain length of alkylsulfonates on their binding constants to pillar[5]arene using a [pillararene] = 0.04 mM at 25 °C. Value for propanesulfonate was extrapolated from [host] = 0.25 mM (see text). (●) Binding constants for alkylsulfonates to β-cyclodextrin taken from ref. [73].

Figure 8 plots the dependence of the binding constant with the alkyl chain length and includes similar results using β-cyclodextrin as a receptor [73]. Quantitative analysis of the thermodynamic parameters involved in the complex formation between surfactant molecules and cyclodextrin can be simplified by considering the process divided into three stages:

(i) Dehydration of surfactants and cyclodextrin This process is entropically favored due to a strong water structuring that hydrates the exposed hydrophobic residue of the surfactant and to geometric constraints within the CD cavity. Water is structured around the surfactant hydrophobic chain, giving rise to a strong network of hydrogen bonds. The amount of water molecules involved in hydration scales linearly with the alkyl chain length, therefore, the linear relationship between the number of carbons present in the surfactant hydrocarbon chain and the micellization free energy, and similar phenomena involving removal of the surfactant chain from the aqueous medium.
(ii) Inclusion of the surfactants in the CD’s cavity. Inclusion takes place with the entry of the surfactant hydrocarbon chain inside the cavity, which is stabilized by Van der Waals interactions. The internal diameter of β-CD allows the loose accommodation of a methylene group.
(iii) Hydration of the inclusion complex. In the last stage, water from the exposed part of the guest is restructured and integrated into the hydration shell of the host:guest complex [74].

Alkylsulfonate binding constants to β-CD increase with the number of methylene groups into the alkyl chain in a non-linear way. The binding constants found for short and very large alkyl chains present lower values than expected due to the fact that the cavity occupation is not complete. This implies that a small amount of water molecules is expelled into the bulk. On the other hand, in the case of large chains, the fact that the binding constants present values lower than those expected would be due to the tolerance of the cyclodextrin cavity to accommodate 6–8 methylene groups.

Figure 8 shows that pillararene is a much more effective receptor for alkylsulfonates than β-CD by a factor of 10^6^. This effect should be ascribed to electrostatic interactions between the negative charge of the guest and the positive ones on the upper and lower rim of pillararene. Note that this interaction is not possible in the case of β-CD as a receptor. The influence of the alkyl chain length on the binding constants to pillararene parallels that observed with β-CD indicating that hydrophobic interactions are playing an important role in the recognition ability of pillararene.

Hydrophobic effects in pillararene recognition are responsible for the different locations of the sulfonate group with respect to the positive upper or lower rim of the host. This different location is reflected by the complexation-induced upfield effect observed in Figure 6-left for hydrogens in alpha position (Ha) to the sulfonate group. Electrostatic interaction in the host:guest complex will compel the sulfonate group close to the trimethylammonium ones in such a way that the distance between the hydrogens Ha of the guest and the aromatic ring of the host keeps constant. However, experimental results indicate that this distance decrease for the following alkylsulfonates: C_8_SO_3_^−^ > C_3_SO_3_^−^ > C_6_SO_3_^−^ > C_5_SO_3_^−^ ≈ C_4_SO_3_^−^. X-ray crystal structure of 1,4-dipropoxypillar[5]arene confirmed that it is a pentagon from the upper view and a pillar structure from the side view. The diameter of the internal cavity was 4.7 Å, which is similar to that of cyclodextrin, allowing the perfect inclusion of methylene chain [75]. The height of pillararene cavity, taken as the distance between the oxygen atoms in the upper and lower rims, is 5.5 Å, allowing accommodation of 4–5 methylene groups. It means that C_5_SO_3_^−^ and C_4_SO_3_^−^ are deeply included in the pillararene cavity in comparison to C_8_SO_3_^−^ and C_3_SO_3_^−^. The smaller alkylsulfonate does not displace a large amount of water from the host cavity resulting in a small hydrophobic effect. On the other hand, three methylene groups of C_8_SO_3_^−^ will be outside the cavity. Their hydration in the host:guest complex will contribute unfavorably to its stability.

## 4. Conclusions

To sum up, we have demonstrated that alkylsulfonates with different chain lengths are effectively bound by a decacationic pillar[5]arene receptor in an aqueous solution with binding constants in the micro/submicromolar range. The formation of the complexes is enthalpy and entropy driven suggesting that ionic, C-H⋯π, van der Waals interaction along with hydrophobic effects contribute to the binding stability. The observed increase in the binding constants as the guest alkyl chain length increases provides strong evidence for the contribution of the hydrophobic effect for the recognition process. This view is supported by the structural NMR studies showing that hydrophobic alkyl chains are deeply included in the aromatic cavity of the macrocyclic receptor. The results obtained herein suggest that cationic pillararene receptors are potentially strong binders for anionic and eventually zwitterionic lipids, and therefore, further studies addressing this class of natural molecules as a guest should be considered due to the potential pharmaceutical applications of these macrocycles.

## Figures and Tables

**Table 1:** Magnitude of the complexation induced chemical shifts (ppm) for host:guest complexes between pillararene and different alkylsulfonates.

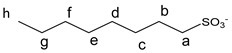 Δδ (ppm)								
	Ha	Hb	Hc	Hd	He	Hf	Hg	Hh
C_8_SO_3_^−^	0.91	1.48	3.27	3.58	2.37	1.46	0.56	0.14
C_6_SO_3_^−^	1.11	1.72	3.7	3.8	2.02	0.98		
C_5_SO_3_^−^	1.24	1.78	3.84	3.85	1.76			
C_4_SO_3_^−^	1.24	1.84	3.33	2.81				
C_3_SO_3_^−^	0.94	1.2	2.16					

**Table 2:** Thermodynamic parameters obtained for host:guest complexes between pillararene and different alkylsulfonates.

	K (M^−1^)	ΔG^0^ (kcal mol^−1^)	ΔH^0^ (kcal mol^−1^)	TS^0^ (kcal mol^−1^)
C_8_SO_3_^−^	(4.79 ± 0.18) × 10^7^	−10.43	−8.90 ± 0.01	1.5
C_6_SO_3_^−^	(1.67 ± 0.02) × 10^7^	−9.91	−8.86 ± 0.01	1.05
C_5_SO_3_^−^	(5.88 ± 0.04) × 10^6^	−9.19	−8.70 ± 0.01	0.49
C_4_SO_3_^−^	(2.63 ± 0.01) × 10^6^	−8.72	−6.60 ± 0.01	2.12
C_3_SO_3_^−^	(7.22 ± 0.02) × 10^3^	−5.24	−4.42 ± 0.01	0.82

## Supplementary Materials

The following are available online at https://www.mdpi.com/article/10.3390/pharmaceutics14010060/s1, Figures S1–S4 show each titration of alkylsulfonates fitted by the “one set of binding sites” model.
