# Supplementary material for: Molecular Recognition by Pillar[5]arenes: Evidence for Simultaneous Electrostatic and Hydrophobic Interactions
Source: Pharmaceutics. 2021 Dec 28;14(1):60. doi: 10.3390/pharmaceutics14010060 (PMC8777861; doi:10.3390/pharmaceutics14010060)
Supplement: Supplementary file 1 [file pharmaceutics-14-00060-s001.zip › pharmaceutics-1489608-supplementary.pdf]

pharmaceutics

Supplementary Materials: Molecular Recognition by Pillar[5]arenes: Evidence for Simultaneous Electrostatic and Hydrophobic Interactions

B. Gómez-González, L. García-Río, N. Basilio, J. C. Mejuto and J. Simal-Gandara

Figures S1-S4 show each titration of alkylsulfonates fitted by the “one set of binding sites” model.

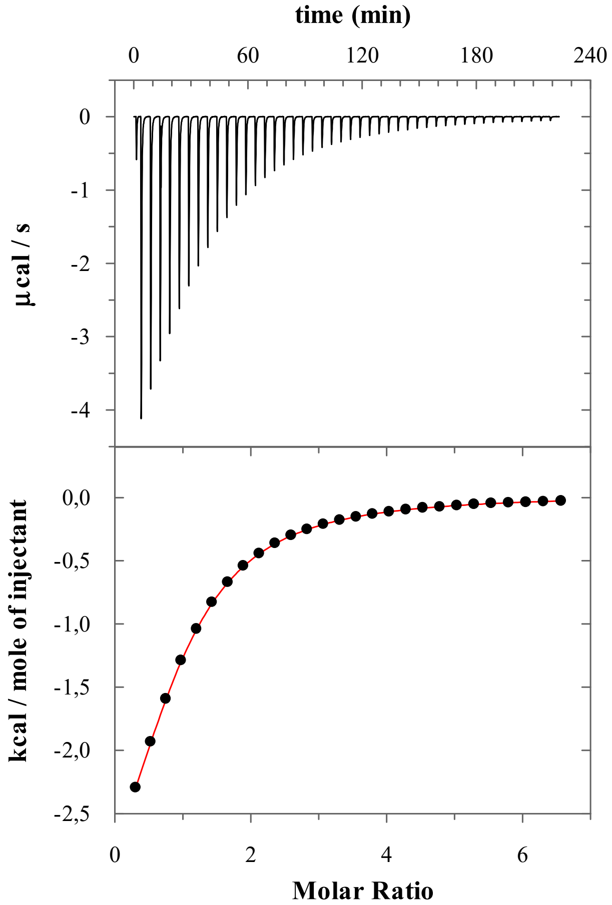

Figure S1. Microcalorimetric titration of propylsulfonate (G) with pillar[5]arene (H) in water at 25ºC. Top: Raw data for the 28 sequential injections (10 µL per injection) of a solution of G (0.5mM) into a solution of H (0.04mM). Bottom: “Net” heat effects obtained by subtracting the dilution heat from the reaction heat, which was fitted by computer simulation using the “one set of sites” binding model.

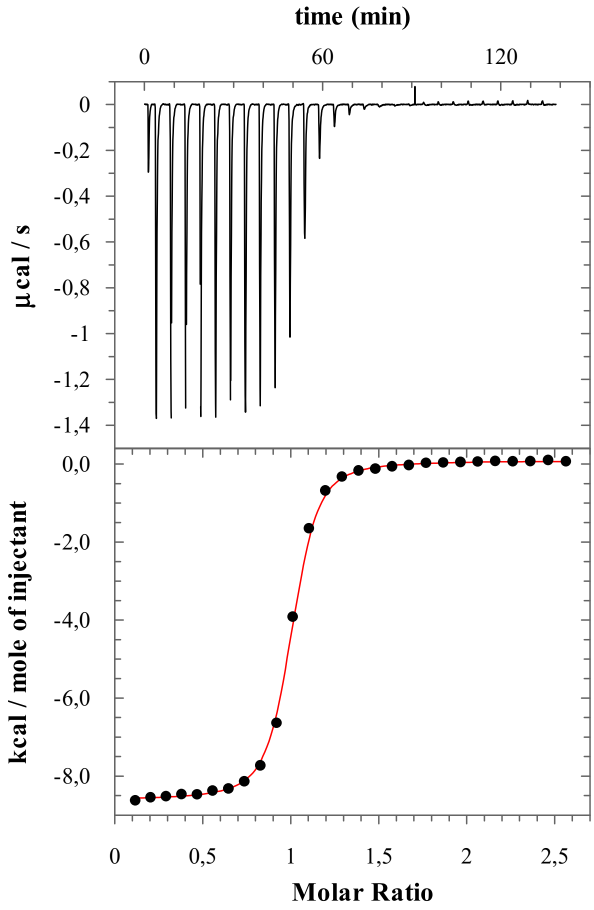

Figure S2. Microcalorimetric titration of pentylsulfonate (G) with pillar[5]arene (H) in water at 25ºC. Top: Raw data for the 28 sequential injections (10 µL per injection) of a solution of G (0.5mM) into a solution of H (0.04mM). Bottom: "Net" heat effects obtained by subtracting the dilution heat from the reaction heat, which was fitted by computer simulation using the "one set of sites" binding model.

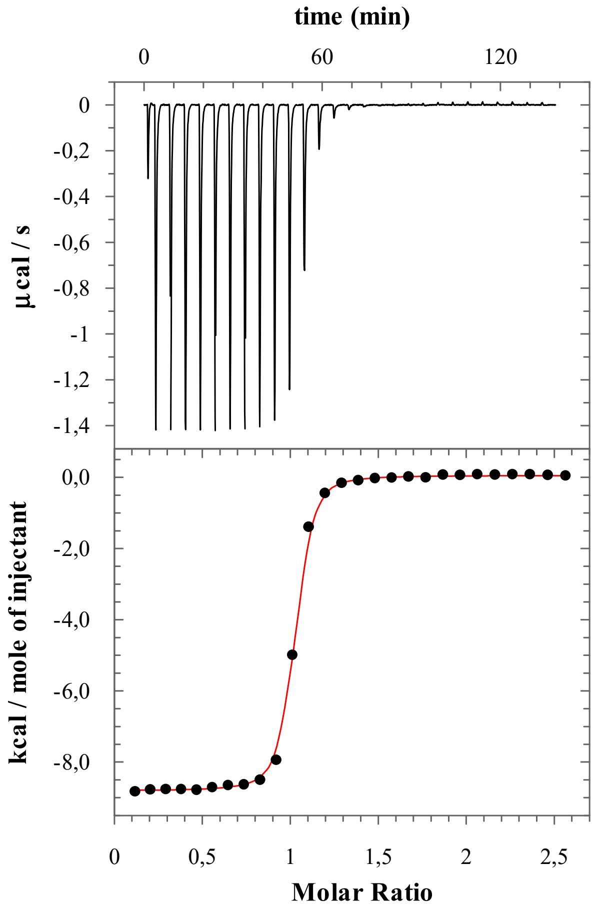

Figure S3. Microcalorimetric titration of hexylsulfonate (G) with pillar[5]arene (H) in water at 25ºC. Top: Raw data for the 28 sequential injections (10 µL per injection) of a solution of G (0.5mM) into a solution of H (0.04mM). Bottom: “Net” heat effects obtained by subtracting the dilution heat from the reaction heat, which was fitted by computer simulation using the “one set of sites” binding model.

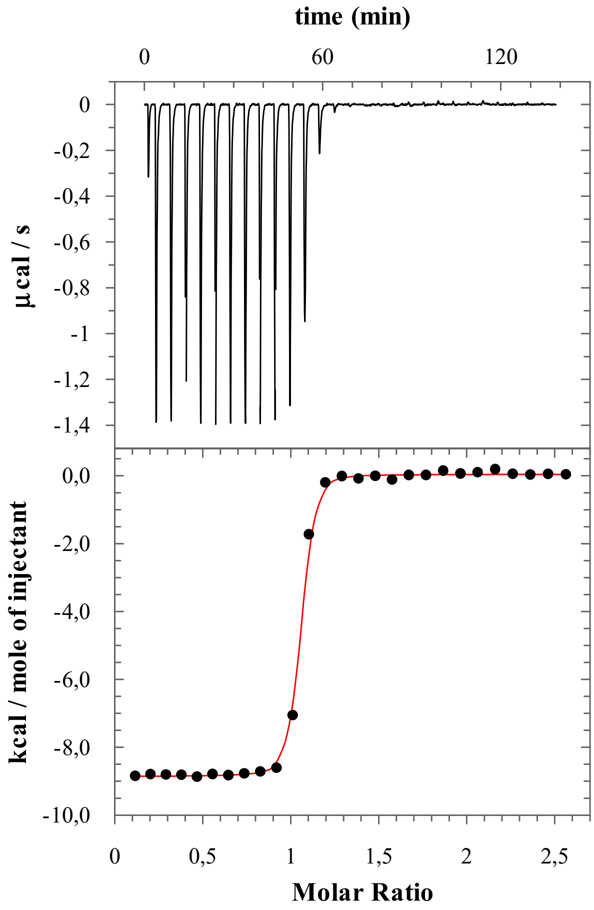

Figure S4. Microcalorimetric titration of octylsulfonate (G) with pillar[5]arene (H) in water at 25ºC. Top: Raw data for the 28 sequential injections (10 µL per injection) of a solution of G (0.5mM) into a solution of H (0.04mM). Bottom: "Net" heat effects obtained by subtracting the dilution heat from the reaction heat, which was fitted by computer simulation using the "one set of sites" binding model.
